# Digital health technology-specific risks for medical malpractice liability

**DOI:** 10.1038/s41746-022-00698-3

**Published:** 2022-10-20

**Authors:** Simon P Rowland, J. Edward Fitzgerald, Matthew Lungren, Elizabeth (Hsieh) Lee, Zach Harned, Alison H. McGregor

**Affiliations:** 1 Haleon (formerly GSK Consumer Healthcare), London, UK; 2 KPMG International Healthcare, Hamilton, Bermuda; 3 Stanford Center for Artificial Intelligence in Medicine, Palo Alto, CA USA; 4 Google Enterprise Health Privacy, San Francisco, CA USA; 5 IP and Technology Transactions, Fenwick & West, Santa Monica, CA USA; 6 Imperial College London, London, UK

**Keywords:** Health policy, Medical ethics

## Abstract

Medical professionals are increasingly required to use digital technologies as part of care delivery and this may represent a risk for medical error and subsequent malpractice liability. For example, if there is a medical error, should the error be attributed to the clinician or the artificial intelligence-based clinical decision-making system? In this article, we identify and discuss digital health technology-specific risks for malpractice liability and offer practical advice for the mitigation of malpractice risk.

The global digital health market is worth approximately 300 billion USD^1^ and is predicted to grow by up to 25% this year. Consequently, medical professionals are increasingly required to use digital technologies such as telehealth platforms, artificial intelligence (AI)-driven clinical decision-making tools, digitally enabled surgical tools, mHealth technologies, or electronic health records (EHRs), as part of care delivery. These technologies hold clear benefits for enabling more efficient, modern care delivery; however, there are significant challenges to implementation, including when and how to use them, how to enable an accurate medical diagnosis in a virtual environment, interpretation and relevance of novel data points from digital devices, the potential for automation bias, appropriate utilization of and engagement with digital disease management platforms, and continuity of care in a digital world. Several of these issues have become apparent through the pandemic due to the hasty deployment of novel technologies as ‘bolt-on’ solutions to address standalone challenges in healthcare delivery, without consideration of the broader healthcare architecture.

The majority of practicing clinicians are not sufficiently trained in how to safely integrate digital health technologies into the clinical workflow before encountering such technologies in practice. The introduction of digital health technologies may therefore represent a risk for medical error and subsequent malpractice liability. Medical malpractice is frequently defined as a physician’s failure to comply with customary medical practice^2^; however, the application of this standard in the context of digital health is challenging. What are the accepted norms for history and examination during a telehealth consult? How should these be documented on electronic systems? When is it safe to offer digital first solutions for disease management? What is the custom for clinicians to ensure continuity of care? If there is a medical error, should the error be attributed to the clinician or the AI-based clinical decision-making system?

In this article, we identify and discuss technology-specific risks for malpractice liability arising from the rapidly growing use of digital health technologies.

## Telehealth

A 2019 survey of 1449 physicians from the American College of Physicians found that the major barriers to adoption of telehealth included difficulties integrating them into the practice workflow (42%), patient access to the technology (36%), concern about potential medical errors (29%), and security and privacy of patient information (23%)^3^. Since then, there has been a rapid increase in use of telehealth with around 23% of all consultations conducted over telehealth in 2020 versus <1% in 2019^4^. This has been driven by the need to avoid physical consultations during the pandemic, more flexible reimbursement models in many major healthcare systems, pandemic-related legislation removing cross-border medical practice barriers between states (US), as well as the introduction of numerous new telehealth platforms that enable access to telehealth for the broader population. The remaining challenges to adoption of telehealth as part of the clinical workflow however remain. Indeed data suggests while the proportion of telehealth consultations has fallen gradually since its peak in mid-2020, it still accounted for 4.5% of all US claims data in July 2021, which is significantly higher than pre-pandemic levels^5^. Concerns over the potential for medical errors stems from the limitations of virtual clinical environments, particularly the inability to physically examine a patient and challenges in communicating in a virtual environment. These limitations are particularly significant at initial consultation when the goal of the consultation is to make a diagnosis, often with no prior medical information.

An analysis by a US-based professional liability provider found that 66% of telemedicine-related claims between 2014 and 2018 were related to misdiagnosis^6^. This is in keeping with findings from Harvard Medical School where a review of cases that had resulted in malpractice claims by experienced clinicians found that 68% were due to failed diagnosis^7^. In contrast, misdiagnosis has been estimated to account for approximately 47% of claims associated with in-person consultations^8^. The consequences were significant with loss of life being the commonest (44%). A high proportion (60%) of cases were settled or awarded to the plaintiff with errors commonly found in documentation or triage. Outside of the US, recent data from the UK has further highlighted the challenges of accurate diagnosis of common conditions when using telehealth platforms, with the number of diagnosed cases of anxiety, depression, coronary heart disease, and stroke approximately 50% lower than expected during the coronavirus disease 2019 (COVID-19) pandemic, at least in part due to significantly increased use of telehealth platforms^9^. In a recent survey of 242 clinicians in Pakistan 69% ‘agreed’ or ‘strongly agreed’ with the statement that there is a ‘lack of regulation to avoid medical malpractice’ and only 29% believed that their medical indemnity would cover telehealth consultations. This level of concern over malpractice risk with telehealth is consistent with anecdotal evidence in the US and other major global healthcare markets^10^. From a population level public health perspective, the risks of patient harm with telehealth platforms must be balanced against the potential benefits such as the potential to assess the patient at an earlier stage in the disease process as a result of easier access to healthcare professionals. Such considerations are important on a population level and should not be lost when establishing ‘customary practice’ for use of telehealth platforms in clinical practice in specific clinical scenarios.

Mitigating the risks of malpractice can be challenging due to the lack of case precedent in telehealth-related malpractice. A 2019 review failed to identify even a single example of a direct to consumer telehealth-related malpractice claim that had been heard in court, suggesting the vast majority are settled outside of court in the US^11,12^. Focussed training and development of clinical skills over telehealth is essential to improve patient safety and thus mitigate the risk of malpractice. A global scoping review of clinical training programmes in telehealth from November 2020 identified a total of 43 different curricula across 11 countries^13^. There was a lack of consensus on the most efficacious techniques for training of clinicians in telemedicine with the authors suggesting a need for greater competency-based, outcome oriented education. In a recently published survey of US-based family physicians, confidence was high in taking medical histories over telehealth (Median Score 5/5 on 5-point Likert Scale); however, it was rated as only 3 out of 5 (Median Score) for other key clinical skills including guiding a patient through the clinical examination over telehealth, managing multiple complaints and language difficulties virtually, and in making decisions remotely on patient logistics, such as transport^14^. Prescribing of controlled medicines over telehealth was the area in which clinicians had the lowest confidence levels (Median rating 2/5) reflecting a high level of perceived risk in this activity. Trainers’ confidence in assessing telehealth skills was variable with virtual communication skills and remote clinical examination skills seen as the most challenging topics, again highlighting the need for greater development of competency-based training and assessment.

## AI-enabled technologies for clinical decision support (CDS) tools

More than 130 different AI-based CDS tools were approved by the Food and Drug Administration (FDA) in the last 5 years for use across a number of different therapeutic areas^15^. Additionally, the FDA has recently created a searchable database of AI-enabled medical devices, although not all such devices in this database are CDS^16^. Healthcare professionals are increasingly asked to consider the data that such CDS tools provide as part of the clinical workflow. But should clinicians always follow the direction of the CDS tool? And if not, are they liable if they ignore the advice and this is later shown to have resulted in harm? Often clinical decisions are made holistically based on the best interests of the patient, and thus decision-making logic can be difficult to fully capture in medical records. These are the types of questions potentially relevant to malpractice risk that clinicians must now consider.

The issue of liability in this situation is complex. Price, Gerke, and Cohen have attempted to create a framework for determining when reliance on an AI system could result in liability^17^. This work identifies two scenarios in which liability might be incurred: (1) when an AI system recommends a course of action that falls within the standard of care, the AI system is correct in making such a recommendation, the physician ignores this recommendation, and the patient suffers a harm because of it; and (2) when an AI system recommends a course of action that falls outside of the standard of care, the AI system is incorrect in making such a recommendation, the physician adheres to this recommendation, and the patient suffers a harm because of it. This work is speculative, and we will have to wait until the case law develops to determine if these predictions hold true. One way in which liability for use of AI-enabled CDS tools might be mitigated is for such tools to be designed to be interpretable. Although some of these tools may function as a ‘black box’, it is becoming increasingly common for such tools to provide some level of explanation for their recommendations, even if only on a post hoc basis. For example, a diagnostic tool intended to help physicians detect the presence of pneumonia from a computed tomographic scan might not only produce just the classification but also a heatmap overlay ‘pointing’ to the area in the image that the AI system identifies as the most relevant for the diagnostic classification^18^. The FDA’s recently released final guidance on CDS notes that certain CDS tools may be exempt from FDA oversight if four criteria are met, including that the software does not acquire, process or analyze medical images, signals or patterns, that it displays, analyzes or prints information normally communicated between healthcare professionals, provides recommendations only to a healthcare professionals (vs specific outputs or directives), and the CDS tool provides the healthcare professional with the ability to independently review the basis of the decision. This fourth criterion regarding the ability to review the basis of the CDS tool’s decision illustrates the benefit of interpretability for certain AI-enabled CDS tools^19^. Furthermore, the FDA has emphasized the importance of interpretability in its recently released guiding principles on good machine learning practice for AI-enabled medical devices^20^.

In some cases, liability is dependent on the judgement of jurors. A recent study involving 2000 US-based adults investigated whether a jury would hold physicians liable in a number of simulated malpractice claims involving AI^21^. The findings suggest that physicians who receive advice from an AI system to provide care aligned to standard protocols may reduce their liability risk by accepting the advice. The data was analysed against Price et al.’s framework revealing that these ‘jurors’ concurred with Price et al. in believing that scenario (1) should result in liability; however, they disagreed regarding scenario (2), suggesting that there might be no liability for the physician in this case^22^. Additionally, the ‘jurors’ further disagreed with Price et al. regarding the scenario in which an AI system recommends a course of action that falls outside the standard of care, the AI system is correct in making such a recommendation (viz. the standard of care is incorrect), the physician ignores this recommendation, and the patient suffers a harm because of it. Price et al. predicted no liability in such a case, however the results were mixed according to the ‘jurors’, indicating that this scenario might in fact result in physician liability.

CDS tools, however, may further reduce risk of liability through result alerting, diagnostic decision support, and electronic tracking, according to the findings of a review of 477 closed malpractice cases from Harvard medical^23^. This study found that the claims in these cases totalling >US$40 million were potentially preventable through use of CDS tools. However, clinicians should be aware of the potential implications of using CDS tools in practice and should be trained on how to use them to protect patient safety and reduce the risk of malpractice. Training should cover technology-specific risks such as decision making in the context of uncertainty with clinical tests^24^.

## mHealth and malpractice liability

mHealth is a broad term that refers to apps and devices with a range of health-related functionalities. We have previously outlined the scope of mHealth, classifying apps and devices according to their functionalities^25^. Commonly, mHealth apps and devices are used to track health-related parameters, analyse such data, and provide personalized health management advice. For example, an Apple Watch and companion app may be used to continuously monitor heart rhythms to identify subclinical signs of ill-health. Data from the Apple Heart Study^26^ suggests that while such technologies may hold significant value in early diagnosis, there is still a ‘false positive’ rate where healthy individuals may be incorrectly diagnosed with a medical condition, causing unnecessary concern. In the aforementioned study 0.52% of individuals were ‘falsely’ notified of an irregular heartbeat, a figure that is significant across an estimated population of approximately 100 million plus Apple Watch users globally. The authors acknowledge that the study was not designed to assess sensitivity and specificity and suggest that the false positives may be due in part to atrial fibrillation being paroxysmal as has been seen in studies of cryptogenic stroke that found differences in the diagnostic yield of atrial fibrillation between loop recorders and 7-day Holter monitor^26^. An incorrectly diagnosed individual may potentially bring a malpractice claim but liability may be unclear, particularly if the monitoring mHealth device has been recommended by a clinician, or the device data has been used as part of the clinical workflow. In another example, an individual may track their menstrual cycle and bleeding through an app, which then analyses the data and provides individual fertility status to the user for the purposes of pregnancy prevention^27^. Such a product may be recommended by a clinician but may fail due to software-related issues outside of their control, again creating uncertainty in terms of clinical liability.

mHealth apps and devices are increasingly popular with consumers as more people seek to become partners in their health choices. A clinician may recommend an mHealth app as a tool to promote self-care and optimize lifestyle management of a medical condition but by doing so they are potentially introducing additional malpractice liability^28^. This is particularly relevant if the clinician cannot justify the advice that is being offered by the app, perhaps because an algorithm is used to provide many different versions of health management advice based on personal data, which may in some cases differ from the appropriate standard of care. In other scenarios clinicians may rely on the data from such mHealth apps and devices to make clinical decisions as part of remote monitoring programs. In this situation, the clinician may be required to make treatment decisions based on unknowingly incorrect information, which arises due to patient input error or device failure. It is unclear whether a clinical decision that results in harm, but which has been made based on incorrect data, would be considered grounds for a malpractice claim. Such a decision will be made on a highly fact-specific basis; for example if the clinician had reason to suspect that the data were incorrect (e.g. the data was a significant outlier, the clinician knew that the patient was unreliable in completing self-report measures, or the clinician was aware of the app’s poor user interface and its being prone to collect erroneous data, etc.), then the clinician will be more likely to be held liable in such an instance. Clinicians may consider having patients sign consent for mHealth apps and disclaim their associated liability.

## Electronic health records

EHRs can be defined as ‘real-time, patient-centred records that make information available instantly and securely to authorized users’^29^. While clinicians can immediately recognize the potential value of EHR over paper-based records, which are generally location specific, include vast amounts of data in non-standard formats from different healthcare sources, and are time-consuming to navigate effectively; in practice the promise of instantly available, relevant clinical information has been difficult to achieve. There are numerous different EHR systems in use even within individual geographic regions and it can take considerable time for clinicians to upskill themselves to use these systems efficiently and effectively. ‘Information bloat’ (excessive note length) can easily occur with EHR and relevant information can be difficult to find if the user is not experienced in navigating the electronic system. EHR contents may also be inaccurate or incomplete due to difficulties in data entry arising from burdensome, rigid documentation requirements or from copy-pasting by individual users^30^. It is well established that EHRs may also lead to clinicians focussing too much on the computer and not enough on the patient, increasing the administrative burden on physicians in routine care, and damaging communication and the doctor–patient relationship. A qualitative review of clinical adaptations to patient communication while using EHR systems demonstrated that practicing clinicians are mitigating the risk of medical error by verbally acknowledging the problem with the patient and repositioning them to optimize communication^31^. Other strategies utilized to optimize communication included participation in task-specific versus general EHR training and maintaining an awareness of expected software updates before implementation.

EHRs control the information available to clinicians so their use may impact on the clinical decision-making process, and therefore change the risk profile of an individual’s practice. Data from an analysis of US malpractice claims from one provider showed that EHR-related claims tripled from seven cases a year in 2010 to 22 cases in 2017/2018^32^. Errors in diagnosis were most common accounting for 1/3rd of the total claims. These were due to either user-related issues such as copy-pasting, incorrect and fragmented data entries, or system-related issues such as failure of EHR ‘alarms’, which might serve as reminders or tools to highlight abnormal results. Other user-related issues associated with malpractice claims included insufficient area for documentation, failure of electronic routing of data, lack of integration between systems or failure to ensure information security. Family medicine and internal medicine were the specialties where most claims were made. Other studies have however not demonstrated any increased risk of malpractice claims with the use of EHR. For example, in a review of malpractice claims at physician’s offices in Colorado, USA, 473 physicians used an EHR. Of the 1569 claim abstracts reviewed, 3% were judged ‘Plausibly EHR-sensitive’, 82% ‘Unlikely EHR-sensitive’, and 15% ‘Unable to determine’. EHR-sensitive claims occurred in 6 out of 633 non-users and 2 out of 251 EHR users and were not significantly different to non-EHR users^33^. An individual’s risk profile for EHR-related malpractice claims depends on both user-specific factors (e.g. individual skill-level in using the specific EHR system) and technology-specific factors. Specialty of clinical practice is also likely to be relevant as the information available on EHRs may be more or less relevant for diagnosis and clinical decision making from one specialty to another.

## Digitally enabled operating rooms and risk of malpractice

Surgery-related claims represent around 25% of all medical malpractice claims in the US, second only to diagnosis related^34^. The majority can be traced back to an aspect of surgeon behaviour during the procedure. The introduction of digital surgical technologies has the potential to significantly impact surgeon behaviour for better or for worse and thus may directly impact malpractice risk. An analysis of the Bloomberg Law database identified 123 malpractice claims involving robotic surgery between 2000 and 2017^35^. Gynaecological surgeries accounted for the majority of claims (62%), followed by urological surgeries (20%), 2 specialties that have been early adopters of robotic surgery. Device failure was cited in only two claims. Thirty percent of these malpractice claims were made during the first year of availability of the robotic surgical system, with the number of claims reducing year on year and highest among early adopters of the technology. Training and experience were identified as a key factor in determining medical malpractice risk with surgical technologies, which is in keeping with findings of previous work from our group demonstrating a significant learning curve with these technologies^36^.

In such cases, the procedure may be recorded via video, which could be used subsequently to identify technical errors or attribute causation to any harm that results from the procedure. Additionally, contactless sensors, including depth, thermal, radio, and audio sensors, are increasingly integrated into surgical equipment. This creates a level of ambient intelligence within operating rooms of the future that will collect huge volumes of data that could inform retrospective analysis of events, for example as part of a malpractice claim review^37^. Data from such sensors are already being used in some cases to automatically assess surgical competence^38^. There is little precedent to guide clinicians working in this space on the risk of malpractice claims associated with new technologies in the operating room or how to mitigate them.

## Cross-border telemedicine and medical malpractice risk

A 2021 survey by the US Cooperative for International Patient Programs (USCIPP) found that of 54 US hospitals surveyed 63% provided telemedicine services to patients and 74% offered teleconsults across international borders. (USCIPP survey) Cross-border telemedicine is associated with additional risks for medical malpractice claims, which may arise due to incomplete knowledge of local standards of care, laws and regulations when advising and treating the patient directly^39^. Telesurgery, which involves a surgeon performing a procedure with instruction from an expert via a digital health platform, is an area of cross-border healthcare delivery that carries several risks for malpractice^40^. This scenario presents many potential questions from a malpractice point of view, such as who is liable and to what extent if there is a technical error due to an incorrect instruction, and what liability would occur if the communication software failed?^41^ Informed consent has also been identified as being a particularly challenging aspect of cross-border telesurgery^42^. As yet there is little precedent to inform mitigation strategies^11,12^; however, the American Telemedicine Association has provided general guidance on cross-border healthcare delivery (https://www.americantelemed.org/policy/).

## Data breaches and cybersecurity threats

The United States Department of Health and Human Services (HHS) defines a data breach as ‘the illegal use or disclosure of confidential health information that compromises the privacy or security of it under the privacy rule that poses a sufficient risk of financial, reputational, or other type of harm to the affected person’. Data breaches due to hacking incidents are the most common, followed by unauthorized disclosures or inappropriate uses by workforce members from within healthcare institutions^43^. The frequency and size of healthcare data breaches are increasing rapidly, leading to an increasing number of lawsuits and regulatory enforcement. In July of 2021 a Florida-based orthopaedic practice is reported to have been sued for US$99 million for a data breach of protected healthcare information secondary to a ransomware attack^44^. Ponemon Institute recently released data in their 2021 report demonstrating that about 67% of patient care organizations have been the subject of cyberattacks, particularly ransomware attacks, during the COVID-19 pandemic. In their report, healthcare respondents reported that ransomware negatively impacted patient care with 71% reporting longer length of stay for patients and 22% reporting an increase in mortality rate.

Up to 50% of internal data breaches have been traced back to negligence on the part of a clinician^45^. Indeed an analysis of 1138 personal health information breaches in the US between 2009 and 2017 showed that most breaches occurred due to human error within organizations, as opposed to from external attacks^46^. In practice, it may be that technical support teams are more likely to be the focus of lawsuits than clinicians, as failures in adherence to best practice standards in data protection, e.g. data encryption techniques may be easier to demonstrate than clinical negligence.

Cyber liability insurance is increasingly included in malpractice insurance packages. Such insurance may provide coverage for costs associated with regulatory fines and penalties, lost income due to downtime, or ransomware fines. Purchasing cyber insurance will become more and more important as digital health platforms are established within clinical practice, but the insurance is not a bullet proof solution, as negligence on the part of the clinician or workforce member’s practices could result in a cyber insurance denial. A healthcare organization could find itself to be the victim of both a cyber breach and cyber insurance claim declination, as Cottage Health did when their insurance determined the hospital’s failures excluded them from insurance coverage^47^. It should be noted that HHS enforcement of data breaches regarding personal health information can only be performed under the Health Insurance Portability and Accountability Act of 1996 (HIPAA), and HIPAA only applies to specific entities: (1) Covered entities, such as healthcare providers or payors; and (2) Business Associates, such as vendors of Covered Entities. But many digital health entities do not fall under either such category, and hence are not subject to HIPAA or its data breach penalties. However, the FTC has recently issued a press release stating that it intends to enforce a decade-old law that can carry hefty financial penalties for data breaches regarding health information, even for entities that are not subject to HIPAA^48^. Furthermore, there are several examples of bills recently submitted for the purpose of broadening the definition of a ‘healthcare provider’ to include big tech companies collecting health data via mHealth devices. These have not yet been successful in altering legislation, but this may change in time.

For a medical error to be considered malpractice, it must fail to comport with customary medical practice. The introduction of digital health technologies into the clinical workflow creates scenarios in which it is challenging to determine what constitutes customary medical practice. There is currently very limited precedent for digital health-related malpractice claims, however errors in diagnosis appear to be the most common cause of claim directly related to the increased use of telehealth platforms, with challenges in communication cited as a potential causal factor. Table 1 summarizes the most frequently cited technology-specific risks for medical malpractice claims. Clinicians are advised to consider their individual risk of malpractice liability before utilizing a digital health technology as part of their clinical practice. Malpractice coverage is likely to vary significantly from policy to policy and from region to region, so clinicians should evaluate their individual situation and consider advice on digital medicine provided by major societies, such as the American Medical Association^49^.

**Table 1:** Summary of most frequently cited technology-specific risks for medical malpractice claims.

Digital health technology	Technology-specific risk(s): most frequently cited	Author comments on malpractice liability
Telehealth	Error in medical diagnosis: ~66% of claims, e.g. due to inability to physically examine a patient and challenges in communicating in a virtual environment	Errors in diagnosis are significant as they are associated with a high proportion of serious injury or loss of life (44% in a US-based study). High proportion claims settled or awarded to plaintiffs (60% in one US-based study). Mitigate through risk informed deployment of technology for specific clinical scenarios only and technology-specific training of clinicians
CDS tools	Scenario 1: CDS rightly recommends within standard of care, but clinician does not follow Scenario 2: CDS erroneously recommends outside of standard of care, but clinician follows	A simulated study of 2000 US-based jurors believed that clinicians should be liable in (1), but perhaps not in (2). Mitigate by addressing ‘black box’ nature of tools, i.e. use tools that provide some level of transparency and reasoning for clinical advice, and through training on uncertainty decision making
mHealth	Error in clinical decision making based on false diagnosis or false data, e.g. from a remote monitoring device	Decision re liability likely made on a highly fact-specific basis. For example, risk increased if the clinician is shown to have a reason to suspect inaccuracy of the data eg. significant outlier, device prone to collection of incorrect data, other clinical explanation
EHR	Error in medical diagnosis: ~33% of claims, due to factors such as poor patient communication secondary to screen focus, ‘information bloat’, challenging user interface, lack of skills in utilizing the system, failure of system ‘alarms’ for abnormal data	In a US claims database EHR-related claims tripled from seven cases a year in 2010 to 22 cases in 2017/18. Both user-specific factors eg. skill navigating the systems and technology-specific factors eg. user interface, or system performance relevant for determination of risk. Training on use of specific EHR platforms is important before deployment. Mitigate risk through clinical behavioural changes such as repositioning to optimize communication
Digitally enabled operating rooms and cross-border telemedicine	Digital surgical platforms lead to challenges in adaptation in terms of technical skills and non technical skills, particularly communication and team work. Telesurgery introduces additional risks for malpractice due to regional and international differences in licensing, regulations and standards of care	Analysis of Bloomberg Law database identified 123 malpractice claims involving robotic surgery between 2000 and 2017. Level of risk dependent on technical skills including surgical skill and experience (documented learning curve). Future risk may be impacted by the growth of ‘ambient recording’ in operating theatres, yielding data that may be used as evidence in context of malpractice claims, and ‘cross-border’ deployment of telesurgery. Mitigate through awareness and understanding of official guidance e.g. American Telemedicine Association

### Reporting summary

Further information on research design is available in the Nature Research Reporting Summary linked to this article.

## Supplementary information

Reporting Summary

## References

[1] Statista. Global digital health market forecast 2025. https://www.statista.com/statistics/1092869/global-digital-health-market-size-forecast/ (2022).

[2] Rich BA (2005). Medical custom and medical ethics: rethinking the standard of care. Camb. Q. Healthc. Ethics.

[3] Frieden, J. Barriers to telehealth adoption remain, survey finds. MedpageToday. https://www.medpagetoday.com/meetingcoverage/acp/79180 (2019).

[4] Weiner JP (2021). In-person and telehealth ambulatory contacts and costs in a large US insured cohort before and during the COVID-19 pandemic. JAMA Netw. Open.

[5] Pifer, R. Telehealth claims drop to lowest level since before pandemic, Fair Health finds. Healthcare Dive. https://www.healthcaredive.com/news/telehealth-claim-lines-drop-to-lowest-level-since-before-pandemic-fair-hea/606298/ (2021).

[6] Uptick in Telehealth Reveals Medical Malpractice Concerns. https://news.bloomberglaw.com/health-law-and-business/uptick-in-telehealth-reveals-medical-malpractice-concerns (2020).

[7] Katz HP, Kaltsounis D, Halloran L, Mondor M (2008). Patient safety and telephone medicine: some lessons from closed claim case review. J. Gen. Intern. Med..

[8] Finnegan, J. Diagnostic errors are top reason for liability claims against primary care doctors, report says. Fierce Healthcare. https://www.fiercehealthcare.com/practices/diagnostic-errors-are-top-reason-for-liability-claims-against-primary-care-doctors-report (2019).

[9] Williams R (2020). Diagnosis of physical and mental health conditions in primary care during the COVID-19 pandemic: a retrospective cohort study. Lancet Public Health.

[10] Alam L, Alam M, Malik AM, Faraid V (2021). Is Telemedicine our cup of tea? A nationwide cross-sectional survey regarding doctors’ experience and perceptions. Pak. J. Med. Sci. Q..

[11] Fogel AL, Kvedar JC (2019). Reported cases of medical malpractice in direct-to-consumer telemedicine. JAMA.

[12] Fogel AL, Lacktman NM, Kvedar JC (2021). Skin cancer telemedicine medical malpractice risk. JAMA Dermatol..

[13] Stovel RG, Gabarin N, Cavalcanti RB, Abrams H (2020). Curricular needs for training telemedicine physicians: a scoping review. Med. Teach..

[14] Venditti SA, Sazegar P, Fuchs LC, Snarskis CE (2022). Family medicine resident and faculty perceptions about the strengths and limitations of telemedicine. Train. Prim..

[15] Wu E (2021). How medical AI devices are evaluated: limitations and recommendations from an analysis of FDA approvals. Nat. Med..

[16] Center for Devices & Radiological Health. Artificial intelligence and machine learning (AI/ML) medical devices. U.S. Food and Drug Administration. https://www.fda.gov/medical-devices/software-medical-device-samd/artificial-intelligence-and-machine-learning-aiml-enabled-medical-devices (2021).

[17] Price WN, Gerke S, Cohen IG (2019). Potential liability for physicians using artificial intelligence. JAMA.

[18] Rajpurkar P (2018). Deep learning for chest radiograph diagnosis: a retrospective comparison of the CheXNeXt algorithm to practicing radiologists. PLoS Med..

[19] Food and Drug Administration. Your Clinical Decision Support Software: Is It a Medical Device? https://www.fda.gov/medical-devices/software-medical-device-samd/your-clinical-decision-support-software-it-medical-device (2022).

[20] Center for Devices & Radiological Health. Good machine learning practice for medical device development. U.S. Food and Drug Administration. https://www.fda.gov/medical-devices/software-medical-device-samd/good-machine-learning-practice-medical-device-development-guiding-principles (2021).

[21] Tobia K, Nielsen A, Stremitzer A (2021). When does physician use of AI increase liability?. J. Nucl. Med..

[22] Price WN, Gerke S, Cohen IG (2021). How much can potential jurors tell us about liability for medical artificial intelligence?. J. Nucl. Med..

[23] Zuccotti G (2014). Reducing risk with clinical decision support: a study of closed malpractice claims. Appl. Clin. Inform..

[24] Neher S, Kapsner LA, Prokosch H-U, Toddenroth D (2021). Design of an interactive web application for teaching uncertainty interpretations of clinical tests. Stud. Health Technol. Inform..

[25] Rowland SP, Fitzgerald JE, Holme T, Powell J, McGregor A (2020). What is the clinical value of mHealth for patients?. NPJ Digit. Med..

[26] Perez MV (2019). Large-scale assessment of a smartwatch to identify atrial fibrillation. N. Engl. J. Med..

[27] Natural Cycles. Natural Cycles birth control. https://www.naturalcycles.com/ (2020).

[28] Wiley, L. F. Malpractice liability and mobile health. https://www.aaas.org/programs/scientific-responsibility-human-rights-law/mhealth-liability-workshop-papers (2018).

[29] Alzu’bi AA, Watzlaf VJM, Sheridan P (2021). Electronic Health Record (EHR) abstraction. Perspect. Health Inf. Manag..

[30] Graber ML, Byrne C, Johnston D (2017). The impact of electronic health records on diagnosis. Diagnosis.

[31] Sieck CJ, Pearl N, Bright TJ, Yen P-Y (2020). A qualitative study of physician perspectives on adaptation to electronic health records. BMC Med. Inform. Decis. Mak..

[32] Leventhal, R. EHRs more frequently factor into medical malpractice claims, study finds. Healthcare Innovation. https://www.hcinnovationgroup.com/clinical-it/news/21095449/ehrs-more-frequently-factor-into-medical-malpractice-claims-study-finds (2019).

[33] Victoroff MS, Drury BM, Campagna EJ, Morrato EH (2013). Impact of electronic health records on malpractice claims in a sample of physician offices in Colorado: a retrospective cohort study. J. Gen. Intern. Med..

[34] Burke, A., Gilmore, S. & Small, M. A dose of insight – surgery risks. White Paper. https://www.coverys.com/knowledge-center/a-dose-of-insight-surgery-risks (2020).

[35] Nik-Ahd F (2019). Robotic urologic surgery: trends in litigation over the last decade. J. Robot. Surg..

[36] Cundy TP, Rowland SP, Gattas NE, White AD, Najmaldin AS (2015). The learning curve of robot-assisted laparoscopic fundoplication in children: a prospective evaluation and CUSUM analysis. Int. J. Med. Robot..

[37] Haque A, Milstein A, Fei-Fei L (2020). Illuminating the dark spaces of healthcare with ambient intelligence. Nature.

[38] Jin, A. et al. Tool detection and operative skill assessment in surgical videos using region-based convolutional neural networks. Preprint at *arXiv* https://arxiv.org/abs/1802.08774 (2018).

[39] Legido-Quigley H, Doering N, McKee M (2014). Challenges facing teleradiology services across borders in the European union: a qualitative study. Health Policy Technol..

[40] Choi PJ, Oskouian RJ, Tubbs RS (2018). Telesurgery: past, present, and future. Cureus.

[41] El-Sabawi B, Magee W (2016). The evolution of surgical telementoring: current applications and future directions. Ann. Transl. Med..

[42] Ayoub CH, El-Asmar JM, Abdulfattah S, El-Hajj A (2022). Telemedicine and telementoring in urology: a glimpse of the past and a leap into the future. Front. Surg..

[43] Seh, A. H. et al. Healthcare data breaches: insights and implications. *Healthcare* **8**, 133 (2020). 10.3390/healthcare8020133 PMC7349636 32414183

[44] Drees, J. 13 patient data breach lawsuits in the past year. https://www.beckershospitalreview.com/cybersecurity/13-patient-data-breach-lawsuits-in-the-past-year.html (2021).

[45] HIPAA Journal. 53% of healthcare data breaches due to insiders and negligence. https://www.hipaajournal.com/53-of-healthcare-data-breaches-due-to-insiders-and-negligence/ (2018).

[46] Jiang JX, Bai G (2019). Evaluation of causes of protected health information breaches. JAMA Intern. Med..

[47] Farrell, E. M. California district court called upon to determine scope of coverage provided by stand-alone cyberinsurance policy. Data Law Insights. https://www.crowelldatalaw.com/2015/05/california-district-court-called-upon-to-determine-scope-of-coverage-provided-by-stand-alone-cyberinsurance-policy/ (2015).

[48] Federal Trade Commission. FTC warns health apps and connected device companies to comply with Health Breach Notification Rule. https://www.ftc.gov/news-events/press-releases/2021/09/ftc-warns-health-apps-connected-device-companies-comply-health (2021).

[49] American Medical Association. Digital medicine liability and risk: what you need to know. https://www.ama-assn.org/system/files/2018-12/playbook-resource-step-5-liability-risk.pdf (2018).

